# Current News

**Published:** 1898-12

**Authors:** 


### Current News.

### PENNSYLVANIA STATE BOARD OF DENTAL EXAMINERS.

The State Board will hold examinations in Pittsburg and Philadelphia on December 6, 7, and 8, 1898.

G. W. Klump, D.D.S.,
*Secretary.*

### NEW YORK ODONTOLOGICAL SOCIETY.

### PRELIMINARY NOTICE.

The New York Odontological Society will celebrate its thirty-first anniversary on Tuesday, January 17, 1899.

On this occasion the Society will hold an afternoon and evening meeting, and J. Leon Williams, L.D.S., D.D.S., of London, has prepared a paper for each session, and will be present to read them.

*Afternoon Paper.*

"On Certain Controversial Questions and Unsolved Problems in Dental Histology and Pathology."

A criticism of the recent paper on the structure of enamel, by Dr. Otto Waldkoff.

Further researches on enamel structure, with a critical review of the paper on tubular enamels, recently presented before the Royal Society of Great Britain by Mr. Charles S. Tomes, F.R.S.

An examination of the special forms of acid-forming bacteria found attached to the approximal surfaces of teeth.

A brief review of the work of Dr. Filandro Vicentini on the fructification of Leptothrix buccalis.

Illustrated by numerous photographs.

*Evening Paper.*

"Which shall it be, the Scientific or the Empirical Method?"

An examination of the present scientific status of the dental profession in America, as shown by its recent literature.

The results not altogether flattering.

Indifference towards scientific research.

The empirical spirit and method.

The scientific spirit and method.

Are we to have a trade or a profession?

The question not yet decided.

The duty of the colleges. The duty of the societies. The duty of the individuals.

The question of patents and secret preparations.

The French Academy of Medicine.

A plea for the formation of a new national organization, with an established fund and State branches, whose function it shall be to promote original research and all scientific work connected with the profession, and to examine and pass judgment upon all inventions, formulæ for remedies, etc. Such an organization, if properly formed and managed, will be certain to prove a wonderful stimulus to progress in all directions, and at a single blow to destroy all quack remedies and useless inventions. An advance of twenty years at a single step.

The unification of the State laws regulating practice.

Shall we go forward or backward?

B. C. Nash,
F. T. Van Woert,
W. W. Walker, *Chairman*,
*Executive Committee.*

58 W. Fiftieth Street.

---

### NATIONAL SCHOOL OF DENTAL TECHNICS.

The next annual meeting of the National School of Dental Technics will meet on the 28th and 29th of December, 1898, beginning promptly at ten A.M. with the address of President G. V. Black.

The partly made up programme is as follows: "The Value of a Graded Course of Study and Uniformity among Dental Schools," by G. V. I. Brown; "Reports of Syllabic Committees; Operative Technics," by T. E. Weeks; "Prosthetic Technics," by N. S. Hoff; "Symposium of Teaching Methods," by W. H. Whitslar, C. M. Wright, and H. H. Burchard; "Steel Technics," by C. H. Wilson; "Teaching Cavity Preparation," by C. N. Johnson. Master of Exhibits, Grant Molyneaux.

Discussions on the papers will be opened by prominent teachers. It is hoped that all interested in the newer methods of teaching in dental schools will be present. A profitable time is promised. Exhibits of class-work will be interesting. The profession is cordially invited to attend.

Meeting will be held in the Club Rooms of the Grand Hotel, Cincinnati, Ohio.

D. M. Cattell,
*Secretary-Treasurer.*

904 Stewart Building, Chicago.

